# A Case of Right-Sided Infective Endocarditis Requiring AngioVac Debulking

**DOI:** 10.7759/cureus.53251

**Published:** 2024-01-30

**Authors:** Danielle Pawlichuk, Micah Pippin

**Affiliations:** 1 Family Medicine, Louisiana State University Health Shreveport (LSUHS), Alexandria, USA

**Keywords:** staph aureus endocarditis, persistent bacteremia, septic shock in endocarditis, iv drug, septic pulmonary emboli, angiovac and infective endocarditis

## Abstract

Infective endocarditis is an uncommon but consequential disease process that occurs after damage to the cardiac endothelium. Management depends on location and infection severity, but it can typically be treated with intravenous antibiotics. Still, in more complex presentations, surgical intervention may be warranted. Here, we examine a case of right-sided infective endocarditis affecting the tricuspid valve in a patient with a history of intravenous drug use. The purpose of this paper is to examine a case of right-sided endocarditis refractory to intravenous antibiotics, resulting in the need for an alternative treatment modality using AngioVAC debulking.

## Introduction

Infective endocarditis is the infection of a native or prosthetic heart valve, the endocardial surface, or an indwelling cardiac device [1]. It is believed that native valve endocarditis develops after injury to the endothelial lining or endocardium, allowing circulating bacteria in the bloodstream to adhere to the vessel wall and form vegetation [1-2]. As bacteria replicate and continue depositing on the vegetation, they become friable and may break off into circulation. Fragmentation of these vegetations can manifest as the clinical features and complications of infective endocarditis, including bacteremia, septic pulmonary emboli, valvular dysfunction and destruction, and other systemic complications [2-3].

## Case presentation

A 23-year-old female with a past medical history of polysubstance abuse, hepatitis C, depression, and anxiety presented to the emergency room with complaints of fever, shortness of breath, and generalized weakness. The patient stated her symptoms began with abdominal distension approximately ten days prior. At that time, she sought medical attention at an outlying facility, was reportedly diagnosed with pneumonia, and was subsequently discharged on oral antibiotics. Afterward, she continued to experience symptoms with progressive worsening over the next three days, prompting her presentation to the emergency room. She denied experiencing any similar symptoms in the past, such as chest pain, abdominal pain, or other symptoms.

**Figure 1:**
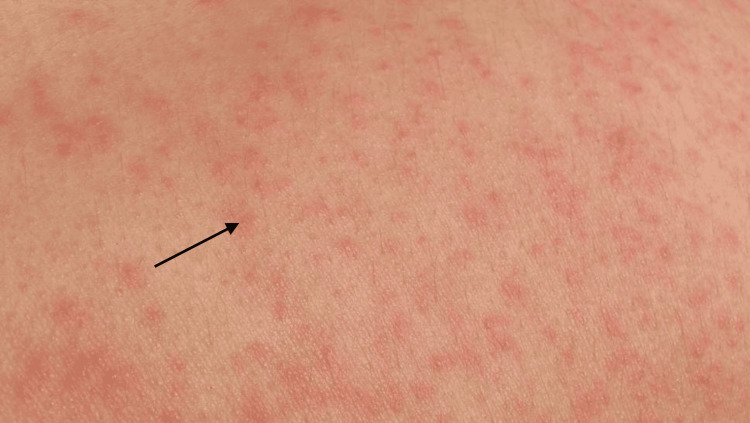
Petechial rash noted on physical examination

On physical examination, the patient was hypothermic, hypotensive, and tachycardic, with a waxing and waning mental status. The patient admitted to a history of daily intravenous fentanyl use in addition to other recreational drugs but stated she had not used drugs in the past two days. Thrush was present in the patient's mouth. The skin examination was significant for a diffuse petechial rash and bilateral lower extremity edema (Figure 1).

**Figure 2:**
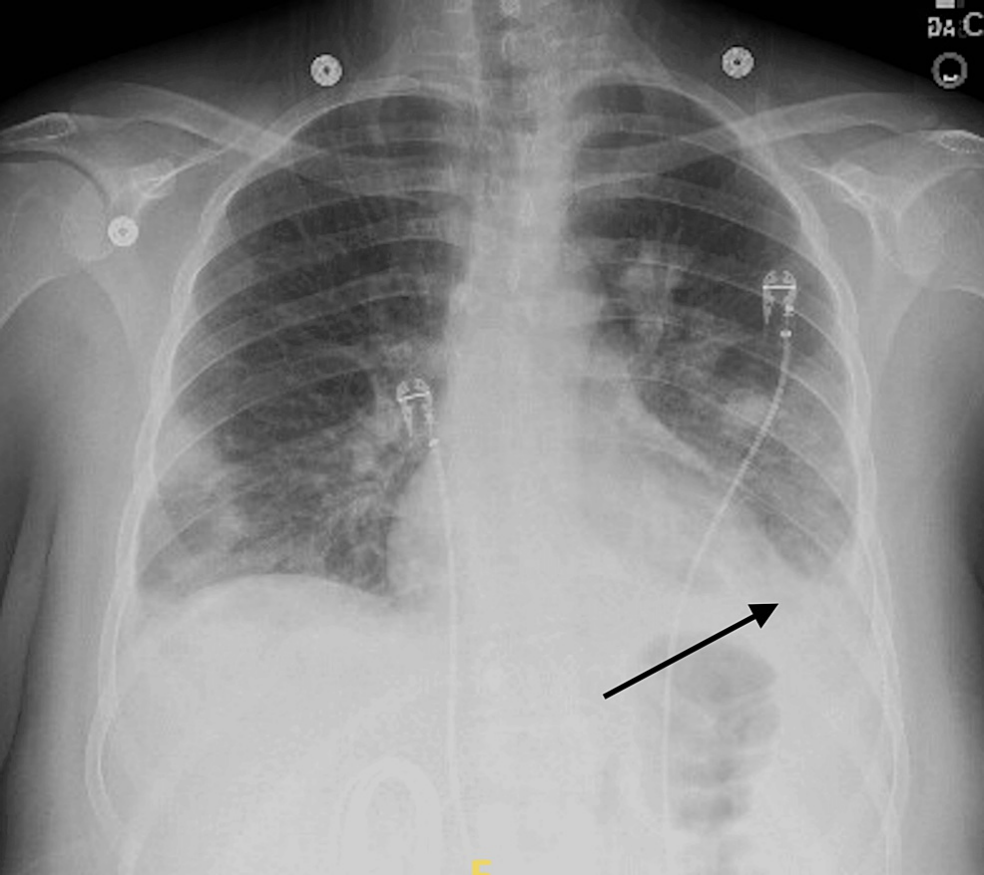
Chest radiography with small left pleural effusion and consolidation

She was given a fluid bolus and started on intravenous fluid hydration per the hospital's sepsis protocol. Levophed was added for additional pressor support. Her complete blood count (CBC) was significant for leukocytosis, bandemia, and thrombocytopenia. Her procalcitonin was elevated at 6.74 ng/mL (normal <0.1 ng/mL). Initial blood cultures grew Gram-positive cocci in four separate samples. Microscopic urinalysis was significant for a urinary tract infection. A urine drug screen was positive for amphetamines and benzodiazepines. C-reactive protein (CRP) and erythrocyte sedimentation rate (ESR) were also elevated at 21.1 mg/L (normal <10 mg/L) and 91 mm/hr (normal 0 to 15 mm/hr), respectively. Chest radiography (CXR) demonstrated bilateral infiltrates with a small left pleural effusion and basilar consolidation (Figure 2). The radiologist suggested the pattern may represent septic pulmonary emboli. A computerized tomography (CT) angiogram of the chest showed small bilateral pulmonary emboli and patchy infiltrates. The patient was subsequently admitted to the intensive care unit for septic shock secondary to bacteremia and a urinary tract infection.

**Figure 3:**
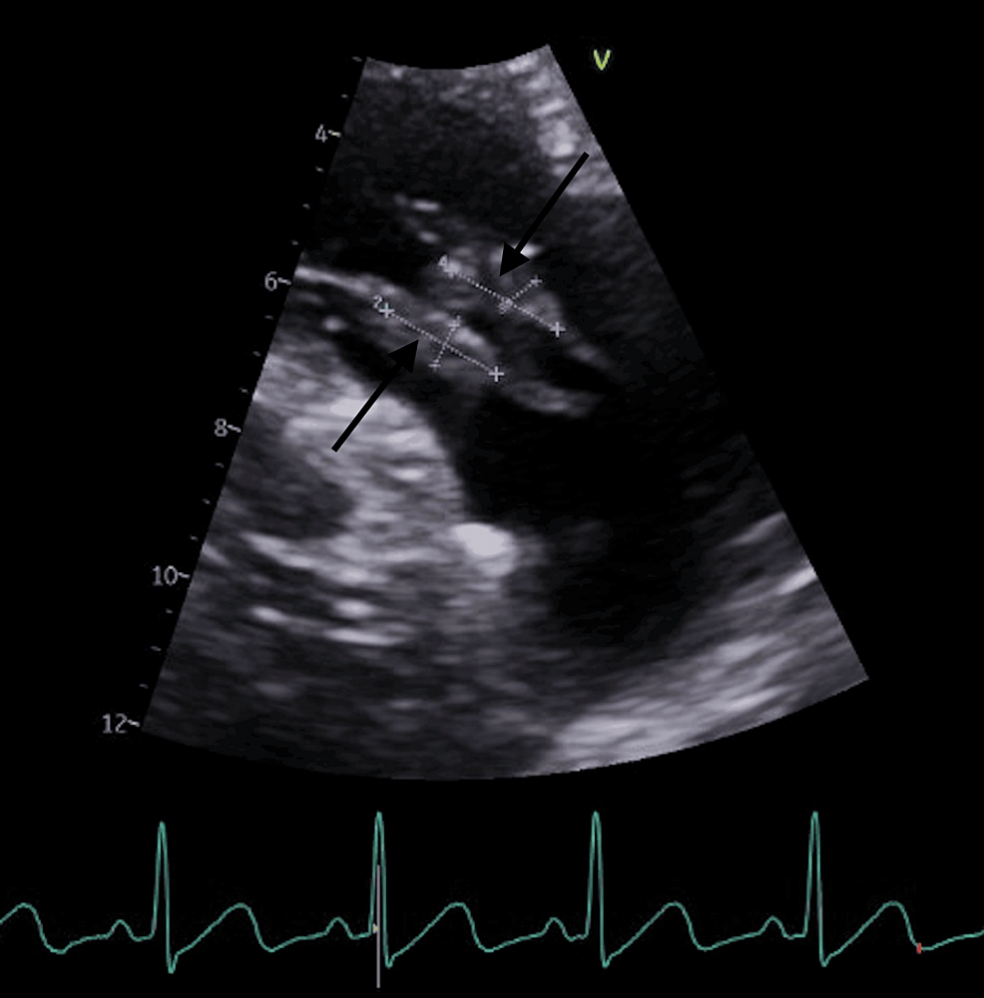
Echocardiogram demonstrating two large vegetations attached to the tricuspid valve

The patient was started on broad-spectrum antibiotic therapy with vancomycin, ceftriaxone, and fluconazole. Repeat blood cultures grew Staphylococcus aureus, which was sensitive to penicillin. The echocardiogram showed two large vegetations attached to the tricuspid valve, measuring 1.6 cm × 0.5 cm and 1.5 cm × 0.6 cm (Figure 3). One of these was large, irregular, highly mobile vegetation. Cardiovascular surgery was consulted; however, the physician could not perform the surgical intervention, so a transfer was recommended.

Due to the patient's complicated history of intravenous drug abuse, her persistent leukocytosis with bandemia, the presence of cardiac vegetation on echocardiography, and Staphylococcus aureus bacteremia, an infectious disease specialist was consulted. The patient was started on intravenous nafcillin every four hours for six weeks, with recommendations for possible AngioVac. After further discussion with cardiology and cardiovascular surgery, it was agreed that the patient would likely benefit from debulking the tricuspid valve vegetation using an AngioVAC device. The size of the vegetation, apparent nonresponse to antibiotic therapy, and septic emboli influenced the decision to proceed with surgical intervention. She was subsequently transferred to a new facility for a higher level of care.

## Discussion

Infective endocarditis is a relatively rare disease, affecting approximately three to ten per 100,000 people [1-2]. Previously, infective endocarditis was thought to have a strong association with congenital heart disease and prosthetic heart valves. However, in recent years, there has been a substantial shift toward instances of infective endocarditis occurring in intravenous drug users [4-5]. The offending organism is most commonly S. aureus, accounting for approximately 70% of infections [4]. Diagnosing infectious endocarditis depends on several factors, including clinical presentation, imaging modalities, and laboratory measurements [6]. The clinical presentation is often variable, ranging from general malaise to fever to sepsis of unknown origin [1-2]. The Duke criteria utilize major and minor clinical criteria to help diagnose and stratify patients with suspected endocarditis. Positive blood cultures and valvular vegetation on echocardiography constitute the major criteria and are diagnostic of infective endocarditis [6]. Minor clinical criteria include fever, a preexisting heart condition or injection drug use, vascular phenomena (such as pulmonary or septic emboli), immunologic occurrences (Roth spots, Osler nodes), and microbiologic evidence that does not otherwise meet major criteria (Table 1) [1-2]. The preferred imaging modality is echocardiography, which helps identify valvular abnormalities or vegetations while assessing overall heart function. If transthoracic echocardiography is indeterminate but there remains a high clinical suspicion for infective endocarditis, transesophageal echocardiography may be implemented with greater than 90% sensitivity [1]. If not treated promptly and appropriately, infective endocarditis can have dire consequences, with mortality rates reaching as high as 25% to 30% [1].

**Table 1:** Duke criteria for diagnosing infective endocarditis

Duke's major criteria	Duke minor criteria
Blood culture is positive for typical microorganisms	Predisposing cardiac lesion
	Intravenous drug use
	Temperature >38 ^o^C (100.4 ^o^F)
	Echocardiogram showing valvular vegetation	Embolic phenomena
		Immunologic phenomena
		Positive blood culture not meeting major criteria

The treatment of infectious endocarditis depends on the infection's severity and etiology. Management usually includes four to six weeks of intravenous antibiotics based on blood culture sensitivities. In patients where infection is uncontrolled or valvular dysfunction leads to heart failure, surgical intervention may be warranted [1,5]. Specific indications for surgical management of right-sided infective endocarditis include right ventricular or atrial failure due to significant tricuspid regurgitation, inappropriate response to antibiotics, large vegetation of more than 2 cm, and the presence of septic emboli despite appropriate antibiotic treatment. Previously, surgical intervention consisted of the replacement or repair of the tricuspid valve. However, the last decade has seen a rise in the AngioVAC procedure. The AngioVAC procedure allows for percutaneous mechanical debulking of the tricuspid valve with a less invasive approach, leading to faster recovery times, shorter hospital stays, and avoiding prosthetic valve placement in intravenous drug users [5]. Possible complications include hematoma formation, retroperitoneal bleeding, and decompensation of tricuspid regurgitation.

The overall prognosis for patients affected by right-sided infective endocarditis is relatively good, with most cases responding to antibiotic therapy and demonstrating a mortality rate of less than 10% [6]. Still, some patients, such as ours, may require surgical intervention, such as AngioVAC debulking.

## Conclusions

The last several decades have seen a rise in the prevalence of right-sided infectious endocarditis, a disease previously associated with implantable devices, and rheumatic heart disease, now a condition commonly seen in intravenous drug users. The clinical presentation can be highly variable, prompting high suspicion for endocarditis in any patient with a history of drug use who presents with unexplained sepsis or fever of unknown origin. While most instances are effectively treated with intravenous antibiotics, more severe or refractory cases may require mechanical debulking using the AngioVAC approach.
